# Transient lingual papillitis: A retrospective study of 11 cases and review of the literature

**DOI:** 10.4317/jced.53283

**Published:** 2017-01-01

**Authors:** Eleni-Marina Kalogirou, Konstantinos I. Tosios, Nikolaos G. Nikitakis, Georgios Kamperos, Alexandra Sklavounou

**Affiliations:** 1 DDS MSc, Department of Oral Medicine and Pathology, National and Kapodistrian University of Athens, Athens, Greece; 2 DDS, PhD, Assistant Professor, Department of Oral Medicine and Pathology, National and Kapodistrian University of Athens, Athens, Greece; 3 DDS, MD, PhD, Associate Professor, Department of Oral Medicine and Pathology, National and Kapodistrian University of Athens, Athens, Greece; 4 DDS, MSc, PhD, Professor, Department of Oral Medicine and Pathology, National and Kapodistrian University of Athens, Athens, Greece

## Abstract

**Background:**

Transient lingual papillitis (TLP) is a common, under-diagnosed, inflammatory hyperplasia of one or multiple fungiform lingual that has an acute onset, and is painful and transient in nature.

**Material and Methods:**

Eleven cases of TLP were diagnosed based on their clinical features. Information on demographics, clinical characteristics, symptoms, individual or family history of similar lesions, medical history, management and follow-up were extracted from the patients’ records. The English literature was reviewed on TLP differential diagnosis, pathogenesis and management.

**Results:**

The study group included 8 females and 3 males (age: 10-53 years, mean age 31.7±12.88 years). Seven cases were classified as generalized form of TLP and 4 as localized form. Nine cases were symptomatic. Time to onset ranged from 1 to 14 days. A specific causative factor was not identified in any case and management was symptomatic.

**Conclusions:**

Although TLP is not considered as a rare entity, available information is limited. Diagnosis is rendered clinically, while biopsy is required in cases with a differential diagnostic dilemma. TLP should be included in the differential diagnosis of acute, painful tongue nodules.

** Key words:**Transient lingual papillitis, fungiform papillary glossitis, tongue, nodules.

## Introduction

The term “*transient lingual papillitis*” (TLP) was introduced by Whitaker *et al.* (1) in 1996 to describe the inflammatory hyperplasia of one to several fungiform lingual papillae that has an acute onset, is painful and transient in nature (1). Similar lesions were, also, reported as “*lingual fungiform papillae hypertrophy*” (2) and “*fungiform papillary glossitis*” (3-5), “*lie bumps or liar’s bumps*” (1,6,7) and “*photocopier’s papillitis*” (8). “*Eruptive lingual papillitis*”, (9) “*eruptive familial lingual papillitis*” and “*eruptive lingual papillitis with household transmission*” (6,9,10) may be included in the spectrum of TLP.

TLP is considered as a common but under-diagnosed disease (11). It was self-reported by 92 (56%) of 163 workers at the Dental School of the Medical College of Georgia, in a study conducted through questionnaires (1). Three variants of TLP have been described, based on their clinical features (1,6,10,12). The localized variant presents with swelling of a single to several fungiform papillae of a solitary lingual area, especially of the tip, the lateral borders and the dorsal surface and may occur in patients of every age with a female predilection (1,11). In the generalized variant a large proportion of the fungiform papillae is involved. During its usual course, a child of a median age of 3.5 years is initially affected and progressively the disease presents in other family members. This form is more consistent with the descriptive terms *eruptive familial lingual papillitis or eruptive lingual papillitis with household transmission* (6,10). Both the localized and generalized forms have an acute onset (1,7,11) and the enlarged papillae may vary in color from normal, erythematous or whitish to yellow, while they rarely appear brown or black, due to staining from food or smoking (13). Moreover, these two clinical patterns may be accompanied by disproportionate symptoms, including pain, burning, tingling or itchy sensation, difficulty in feeding, sensitivity to hot foods (1,6,10,11) and, in cases with familial transmission, hypersalivation and occasionally fever and lymphadenopathy (6,10). Symptoms typically resolve after a few hours or 1 to 4 days (1,7,11), while they may last for 1 to 3 weeks, when diffuse lingual inflammation coexist (11). Biopsy is not required for the final diagnosis (10,11), but in cases where it was performed microscopic examination showed an inflamed fungiform papillae with minimal spongiosis and neutrophils infiltration of the epithelium (1,10,12). Taste buds that are normally present in fungiform papillae were not detected (1,10,12). The papulokeratotic variant of TLP is characterized by chronic, generalized tongue involvement with painless, whitish or white-yellow in color enlarged fungiform papillae, histologically corresponding to parakeratosis (12).

TLP is not considered a rare disease, but only few case reports or case studies are found in the literature (1,2,6,8-18). The aim of the present study is to report 11 new cases of TLP and to review the English literature on its differential diagnosis, pathogenesis and the appropriate management.

## Material and Methods

This is a retrospective study on 11 cases of TLP diagnosed and managed between the years 2009-2014 by three members (K.I.T, N.G.N and G.K) of the Department of Oral Medicine and Pathology, Faculty of Dentistry, National and Kapodistrian University of Athens. Data extracted from patients’ records included sex and age of the patients; time to presentation; symptoms; individual or family history of similar lesions; history of recurrent aphthous ulcerations, herpetic stomatitis, allergic reaction or recent oropharyngeal infection; medical history, in particular concerning systemic diseases and medication, smoking habit, recent blood examination; clinical presentation; management and follow-up. All patients at the time of their initial examination gave written consent for the future use of their data for study. This study was approved by the Research Ethics Committee of the School of Dentistry, National and Kapodistrian University of Athens (NKUOA code number 289).

## Results

**Figure 1:**
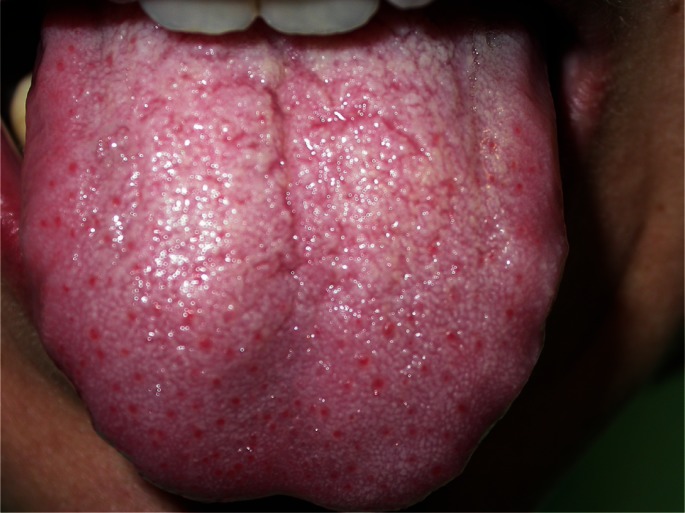
Multiple erythematous enlarged fungiform papillae on the anterior tongue dorsum (#7).

**Figure 2:**
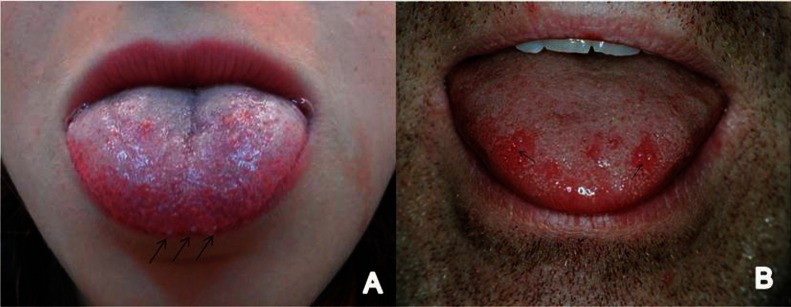
Localized form of TLP affecting A) both sides of the tip of tongue (#1) and B) the anterior tongue dorsum (#2).

 Table 1 summarizes the main clinical features of our cases. There were 8 female and 3 male patients and the mean age was 31.7 years (range 10 to 53 years, SD=12.88). Seven cases were classified as generalized variant (Fig. 1) and mostly involved the ante-rior lingual dorsum and the tip of tongue. In 4 cases few enlarged fungiform papillae were recognized in one or two lingual sites (Fig. 2 A,B). One of those cases was considered as a papulokeratotic variant (case no #8), but as biopsy was not performed, it was classified as localized TLP. Symptoms were present in most cases (81.8%) and included pain, difficulty in eating, especially spicy or acidic food, burning and tingling sensation, xerostomia and dysgeusia. Time to onset ranged from 1 to 5 days, with the exception of case #5 where it was reported to be 2 weeks. Two patients (cases #7 and #10) had used anti-inflammatory agents, antiseptic mouthwashes or an antifungal gel for a few days, without self-reported improvement.

**Table 1:**
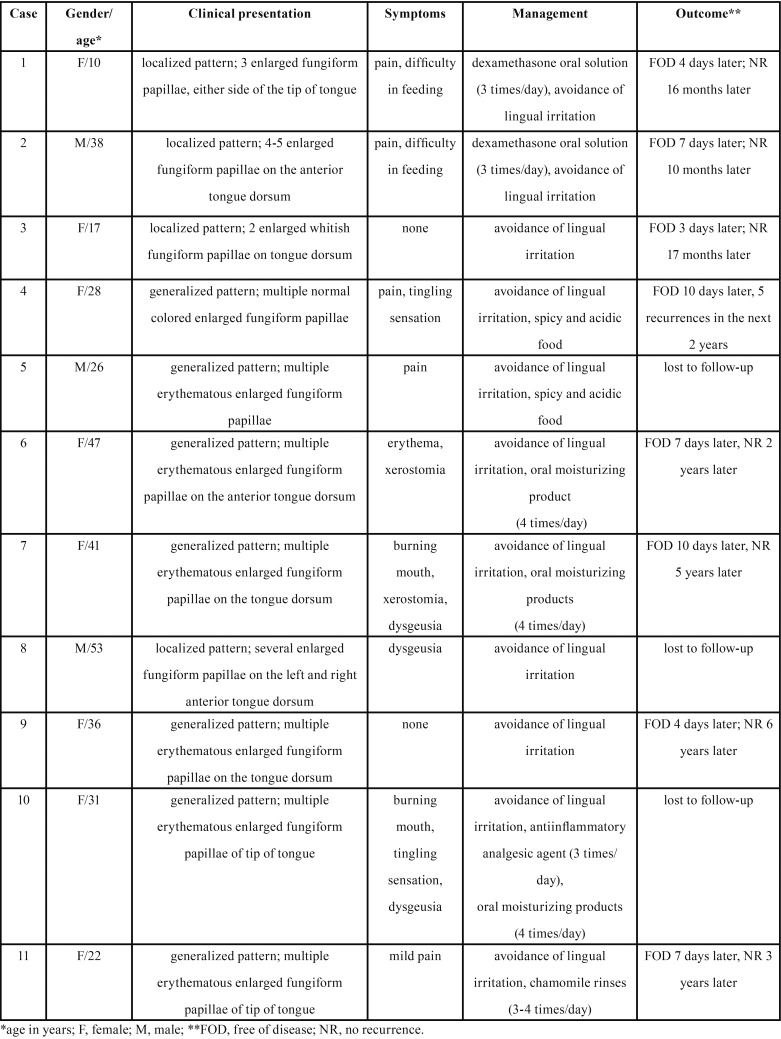
Demographics, clinical presentation, management and outcome of the 11 TLP cases.

None of the patients could remember the occurrence of similar lingual lesions in the past or in other members of their family, except for a high-school student (case #3), who reported that similar lesions had reappeared twice during last year, both times in conjunction with school examinations. Two patients (cases #7 and #11) gave a history of recurrent aphthous ulcerations, while patient in case #2 reported suppurative tonsillitis a month before the appearance of the tongue lesions. None of the patients had a history of herpetic stomatitis or oral allergic reaction, while all denied regular use of antiseptic mouthwashes. Three patients (cases #2, #4 and #8) reported “geographic tongue” that was present at the time of examination in one of them (case #2). Habitual lingual trauma was reported by 2 patients (cases #7 and #10), although in 3 more patients (cases #1, #3 and #11) a diffuse erythematous area on the tip of tongue was indicative of tongue thrusting. In case #1 tongue thrusting on an upper fixed orthodontic appliance was considered plausible. Medical history was not contributory in none of the cases. Patients reported systemic diseases, including familial hypercholesterolemia (case #1), thyroid diseases (cases #4-#6), hypertension (case #8) and β-thalassemia trait (cases #4 and #10). Two patients were smokers (cases #7 and #8) and 1 patient (case #8) reported low blood zinc levels.

**Figure 3:**
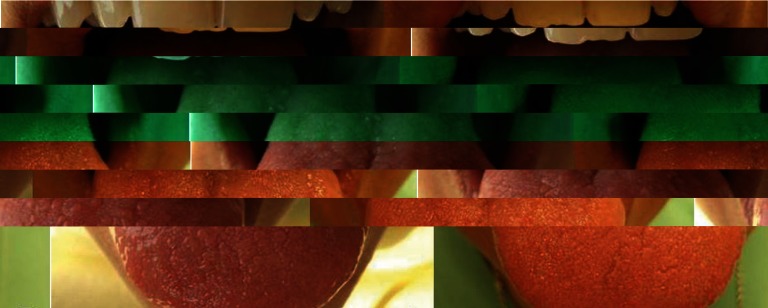
Generalized form of TLP on the tongue dorsum A) at initial examination and B) partially ameliorated 7 days later (#4).

Management of TLP was symptomatic therefore in asymptomatic patients no treatment was prescribed. Patients reporting symp-toms were advised to avoid tongue friction and irritating foods that could exacerbate their symptoms. In cases #1 and #2 where persistent pain caused difficulty in feeding oral rinses with a dexamethasone solution were prescribed, while in patients #6 and #7 that complained for coexisting xerostomia, oral moisturizing products were, additionally, suggested. Fungiform papillae’s enlargement resoluted in 3 to 10 days, while among the 8 cases with available follow up, recurrences were reported only in one patient (case #4), who had initially presented with a diffuse variant of TLP (Fig. 3A,B). In this patient, 5 relapses were seen in 2 years of follow-up.

## Discussion

The cases presented herein were consistent with TLP. Although there are no clear diagnostic criteria for TLP, biopsy is not required for the final diagnosis (10,11). A system proposed for the diagnosis and classification of TLP (3) that is based on color, size and location of the fungiform papillae, is not easily applicable to clinical practice and does not take into account factors such as the symptoms and resolution of the lesions, and normal diversity of fungiform papillae (4).

In our study there was a female preponderance (72.7%), 6 out of 11 patients were older than 30 years, and only two juveniles aged 10 and 17 years, respectively, were found. In previous studies no marked gender preference (6,10-12,16) and a female preponderance (1) are reported, while most patients are in the 1st to 4th decades of life (1,6,10-12,16).

The differential diagnosis of the localized variant of TLP includes reactive oral lesions, such as fibrous hyperplasia, giant cell fibroma and pyogenic granuloma, but in contrast to those lesions, TLP regresses, as was evident in our cases. The generalized enlargement of the fungiform papillae seen in the generalized variant of TLP represents a characteristic finding of scarlet fever that usually manifests in children and is caused by group A (beta-hemolytic) *Streptococcus* (7). Tongue dorsum in scarlet fever initially demonstrates a white coating dispersed with hyperemic enlarged fungiform papillae causing the characteristic “strawberry tongue” (7). “Strawberry tongue” is also seen in strep mouth that unlike scarlet fever is not accompanied by skin rash, as well as Kawasaki disease or mucocutaneous lymph node syndrome, an idiopathic, acute, febrile, multisystem disorder of children, which shares common clinical manifestations with scarlet fever (7). Furthermore, enlarged fungiform papillae were a feature of *psoriasiform fungiform hypertrophy* in 3 patients with a history of psoriasis that developed guttate psoriasis following streptococcal pharyngitis (16). They were also seen in kidney transplant patients receiving cyclosporine A, where they were associated with either change in the microbial flora or poor oral hygiene (2,17) and with increased risk of graft rejection (2). It is not clear whether the lingual enlargement regresses with discontinuation of cyclosporine (2). In psoriasis and cyclosporine A uptake, it is hypothesized that the fungiform papillae do not in fact enlarge, but as the filiform papillae are lost, they protrude and become more apparent (7). A similar phenomenon may be also seen in iron deficiency anemia, erythematous candidiasis and patients receiving chemotherapy (7). Seven of the cases presented herein were consistent with the generalized variant of TLP, but in contrast to previous reports (6,10) no family transmission was reported, while none of the above stated diseases were diagnosed.

Chronic lingual papulosis is considered as the lingual counterpart of the inflammatory papillary hyperplasia of the palate (7). It affects adults and presents as multiple, painless, localized or diffuse, normal colored, enlarged, mainly filiform papillae on the tip or dorsal surface of the tongue (7). Histologically, fibrous hyperplasia is observed, while, in one case, taste buds were also noticed, indicating that the lesion originated from the fungiform papillae. Finally, TLP should be differentiated from diseases manifesting with multiple nodules on the dorsal tongue, including the epidermal nevus syndrome, Bowen papillomatosis, acanthosis nigricans, exophytic form of median rhomboid glossitis, neurofibromatosis, tuberous sclerosis, amyloidosis, lipoid proteinosis, lepromatous leprosy and Cowden syndrome (7). Those nodules are asymptomatic and do not resolve spontaneously, therefore in equivocal cases a biopsy may be indicated. One of our cases was clinically consistent with this variant, but as no biopsy was indicated we chose to classify it as a localized variant.

The etiology of TLP is unknown and probably multifactorial, as it can be hypothesized by the variable and non-specific histological findings (12). It is related to acute or chronic mechanical trauma, compulsive lingual movements because of local irritating factors, such as sharp-edged teeth or restorations, orthodontic appliances or increased calculus on the anterior teeth (1,7,11). Other possible factors include stress, lack of sleep, poor nutrition, geographic tongue (1,11), thermal injury (7), excessive smoking and alcohol uptake (12), consumption of spicy or acidic foods (1), allergy to foods, oral hygiene products or photocopier’s toner (1,8,11), as well as gastrointestinal disorders and hormonal changes during menstruation or menopause (1,11). As TLP is more common in patients with history of atopy, it may also represent a local atopic reaction to heat or irritating foods (3). Often, though, TLP is considered idiopathic (1), while it is also regarded as a relapse in adults of eruptive familial lingual papillitis or *eruptive lingual papillitis with household transmission* that occurred during childhood (6,10). In the present cases, possible triggering factors included the chronic lingual irritation on the orthodontic appliance, the habitual lingual trauma, stress or the coexistence with geographic tongue. In one case, although the patient reported recent suppurative tonsillitis, neither history nor clinical examination were consistent with strep mouth infection.

Infectious agents, particularly viruses, is implicated in the pathogenesis of both TLP and eruptive familial lingual papillitis or *eruptive lingual papillitis with household transmission*, but is not documented (6,10). Immunohistochemical investigation for human papillomavirus types 6 and 11 and herpes simplex virus (HSV) type 1 and 2 (1), as well as the histochemical investigation for fungi and parasites in biopsy specimens were all negative in TLP (1,10,12). In a recent publication, though, Krakowski *et al.* (18) described a case of TLP, where the presence of HSV type 1, was confirmed by direct lesional viral culture, in a patient with Kawasaki disease.

Management of TLP is symptomatic and aims to relieve symptoms (1,11). In painful cases local anesthetics, topical corticosteroids, coating agents, saline mouthwashes and combination of antihistamines with aluminum hydroxide or magnesium hydroxide suspension for topical use have been administrated, and eating of cold foods has been recommended. The use of analgesics such as paracetamol or ibuprofen does not affect the duration and intensity of symptoms (6), while there is no consensus on the usefulness of topical antiseptics (1,6). Patients are also recommended to avoid irritating chewing gums, candies or oral hygiene agents (13). In our cases, divergent therapeutic approaches were decided, all of them conforming to the various treatment modalities proposed in the literature. Most approaches achieved a symptomatic relief.

TLP may relapse (10,11), most commonly its papulokeratotic variant (12). In recurrences the investigation of the possible trigger factors, especially trauma or allergens, is mandatory (11). The available follow-up period in our cases was not adequate for conclusions to be drawn regarding relapses.

TLP is a multifactorial, underdiagnosed disease, occasionally painful. Its recognition and differential diagnosis from other diseases manifesting with lingual nodules helps in avoiding unnecessary diagnostic workup and treatments, while the investigation and identification of possible triggers contributes to relapses’ prevention. Description of more cases will improve our understanding of the disease’s pathogenesis and appropriate management.
